# ‘We Needed a Hell of a Lot More Support, the Emotional Side of It, the Physical Side of It. Every Side of It, We Just Didn't get It.’ A Qualitative Study Exploring the Lived Experiences of Healthcare Services Following Discharge for People With a Total Laryngectomy and Their Families

**DOI:** 10.1111/1460-6984.70275

**Published:** 2026-06-18

**Authors:** Laura‐Jayne Watson, Linda Sharp, David W. Hamilton, Vicky Thornton, Joanne M. Patterson

**Affiliations:** ^1^ University of Liverpool Liverpool UK; ^2^ South Tyneside and Sunderland NHS Foundation Trust Sunderland UK; ^3^ Population Health Sciences Institute, Newcastle University Centre for Cancer Newcastle University Newcastle upon Tyne UK; ^4^ Head and Neck Surgery Freeman Hospital Newcastle upon Tyne UK; ^5^ Institute of Population Health University of Liverpool Liverpool UK; ^6^ School of Allied Health Professions & Nursing, Institute of Population Health, Liverpool Head and Neck Centre University of Liverpool Liverpool UK

**Keywords:** healthcare, laryngectomy, lived experience, qualitative

## Abstract

**Introduction:**

People with a laryngectomy (PwL) and their families commonly require sustained support from healthcare professionals. But the needs of PwL and their families once they return home following hospital discharge are not well understood. Moreover, community healthcare professionals are not specialists in laryngectomy; they work in pressurised environments and lack time and access to education and training. This could impact safety of care and overall adjustment to life after laryngectomy. To better support healthcare professionals in the community to offer a more tailored approach, we first need to understand the needs of PwL and their families.

**Aim:**

This study aimed to understand the lived experience of healthcare services following discharge, including how services could improve, from the perspective of PwL and their families.

**Methods:**

Semi‐structured dyadic and individual qualitative interviews were conducted with PwL±family members. Participants were recruited purposively by clinicians from three head and neck centres in the UK. Interviews were audio‐recorded, transcribed verbatim and analysed using Braun and Clarke's approach to reflexive thematic analysis.

**Results:**

Nineteen interviews were conducted with seventeen PwL and thirteen family members. Three inter‐related themes were established: ‘Immediate Practical and Emotional Demands of Post‐Discharge Life, ‘I had no idea what to do…you don't know anything. What do I do?’; ‘Learning to Cope and Find Acceptance in Everyday Home Life, ‘It was just a matter of coming to terms with the fact that socially and physically I was not the same person I was… I cannot change this, so I don't worry about it, I just accept it.’; and Navigating Healthcare Services in the Post‐Laryngectomy Journey, ‘I understand it's, you know, it's exceptionally difficult to put it all together because there are so many different things…so many different departments all involved in everything.’

**Conclusion:**

This study has highlighted a greater need for better support and intervention for PwL, and their families once discharged home after laryngectomy. Improved collaboration between healthcare professionals and services is needed, as well as better laryngectomy education and training for community healthcare professionals. Future research should focus on exploring how best to achieve this.

**WHAT THIS PAPER ADDS:**

*What is already known on this subject*

- People with a laryngectomy and their families experience devastating permanent changes to everyday life because of the long‐term side effects of surgery. As such, they often require ongoing care following hospital discharge, however their needs are not well understood.

*What this study adds to existing knowledge*

- This study improves understanding of the specific needs of people with a laryngectomy and their families following discharge after laryngectomy. Overall, people feel alone, anxious about equipment and safety and concerned about the need to carry out specialised care at home without specialist support. As such, they have identified a need for better collaboration between healthcare professionals and services involved in their care after laryngectomy.

*What are the potential or actual clinical implications of this work?*

- There is a clear need for ongoing education and training for community healthcare professionals to build confidence and competence in managing people with a laryngectomy, particularly in non‐specialist settings. Strengthening communication between acute, primary, and community care teams is also essential to ensure continuity of care and reduce gaps in service provision. Clinicians could seek to establish these networks within their local teams.

## 1 Introduction

Laryngectomy results in life‐changing consequences for individuals and their families. Long‐term changes to everyday functions have been well‐documented in the literature over the last 30 years (Bozec et al., 2020; Hilgers et al., 1990; Oozeer et al., 2010; Pereira Da Silva et al., 2015). These include permanent loss of natural voice, and life‐long changes to swallowing, breathing and appearance, including the psychological impact this has, for example, disruption to family and social activities. The literature consistently demonstrates how loss of natural speaking voice reshapes identity and communication, limiting participation in everyday interactions, altering family roles and negatively impacting quality of life (Eadie and Bowker, 2012; Sharpe et al., 2019). Changes to swallowing adds further complexity, requiring continuous adaptations to daily living and impacting psycho‐social function (Coffey and Tolley, 2015).

PwL and their families require vital ongoing support from healthcare services following discharge home after surgery. This should be provided in the ‘optimal setting’ (Alderwick and Dixon, 2019) to ensure that people are able to adjust and accept life after laryngectomy. The optimal setting for providing care to people with long‐term health conditions, such as laryngectomy, is a topic currently being discussed at policy level. In the United Kingdom, the Darzi review, and the subsequent NHS long term plan highlights the shift from hospital to home as a key priority (Alderwick and Dixon, 2019, Darzi, 2024). Supporting people to manage their long‐term health needs at home is increasingly viewed as the most appropriate and sustainable setting for this type of care. Additionally, the uptake in enhanced recovery programmes to get people out of hospital sooner (Magos et al., 2022; Wilson et al., 2022), puts the emphasis on healthcare interventions being provided in community settings. This puts pressure on non‐laryngectomy specialist healthcare professionals who are predominantly community‐based but often lack specialist knowledge and skills to manage PwL at home (Darr et al., 2012; Todd et al., 2014). Community‐based healthcare professionals also have inadequate access to education and training (Senior et al., 2021), despite the recognised need (WHITCROFT 2016). Watson et al. (Watson et al., 2025) found that community‐focused laryngectomy education and training resources vary markedly in their methods, content, delivery, and evaluation. Such inconsistency undermines standardisation, making it difficult for healthcare professionals to acquire the reliable, transferable knowledge and skills required to deliver community‐based laryngectomy care (Watson et al., 2025).

Previous research—albeit limited—supports this, with PwL and their families perceiving that they have more knowledge and skills than non‐laryngectomy specialist healthcare professionals to manage their needs (Bickford et al., 2018; Dooks et al., 2012). This is illustrated by survey findings from one centre in France, reporting that (Antin et al., 2021) less than half of laryngectomy participants considered community healthcare professionals to be adequately trained—an indication of a significant and ongoing deficit in community‐based expertise. Additionally, concerns have been raised generally by people with all sub‐sites of head and neck cancer (Checklin et al., 2025), and their families about access to specialist services and support at home from community healthcare professionals, making them feel that they lack control over their care (Watson et al., 2022; Wilson et al., 2022) and have at least one unmet supportive care need (Jansen et al., 2018), for example, local access to support for fatigue management (Foley et al., 2022). This is worrying for PwL, particularly given the impact this has not just on functional management such as airway/breathing; but also, on psycho‐social aspects of care (Offerman et al., 2015). Up to 40% of PwL report social withdrawal and 57% of spouses state that they experience psychological distress linked to changes to their social lives and intimacy (Summers, 2017). As a result, around 20% of spouses report anxiety or depression (Summers, 2017). Despite these concerns, there is a lack of understanding of the lived experience of healthcare following discharge for PwL and their families. A recent study by Checklin et al. (Checklin et al., 2025) explored healthcare experiences among people with head and neck cancer living with communication changes, revealing the system to be overwhelming, complex, and often traumatic. Empathetic professionals and timely specialist input were identified as important in reducing these negative experiences. While this work offers important insights into the broader challenges faced by individuals with communication impairments, these experiences are not specific to PwL and their families. Improved understanding of this under‐researched topic would provide healthcare professionals with an insight into what knowledge and skills are needed to better support PwL and their families at home.

This study aimed to explore PwL and their family members lived experience of healthcare services following discharge, including how services could improve.

## 2 Materials and Methods

### 2.1 Study Design

This cross‐sectional qualitative design used semi‐structured dyadic and individual interviews with PwL ± a family member, where available. The Reflexive Thematic Analysis Reporting Guidelines (RTARG) (Braun V, 2024) were used to guide reporting of the methods, results and discussion. Semi‐structured interviews were conducted in parallel with analysis by the lead author (xxx).

### 2.2 Methodology

Constructivist grounded theory informed the study's approach to qualitative data collection and analysis. Constructivist grounded theory is a flexible and reflexive qualitative approach to generate theory grounded in empirical data (Charmaz, 2009, Charmaz, 2014). This methodology does not seek to test pre‑existing theories; instead, theory is constructed through iterative analysis of participants’ accounts, acknowledging the co‑construction of meaning between researcher and participants and the influence of context and interpretation (Mills et al., 2006). This approach was considered appropriate as it allows existing clinical and theoretical knowledge of laryngectomy care to inform the analysis, while maintaining a clear commitment to reflexivity and transparency throughout the research process.

### 2.3 Topic Guide

A topic guide was used flexibly throughout the interviews so that they could be adapted for the target participant that is, PwL or a family member; and to ensure participants could discuss issues in an order that was natural to them (see supplementary material). The topic guide was based on the outcome of a previous environmental scan (Watson et al., 2025), available literature, lived experience of patients who contributed to stakeholder work (laryngectomy groups) and the research patient and public involvement and engagement (PPIE) group. Topics included questions directed towards the research aim, for example, ‘Can you tell me about your transition from hospital to home?’. The guide evolved naturally and iteratively as interviews progressed (Clarke and Braun, 2013). This ensured that new issues raised during data collection and interim analysis were explored in sufficient depth in subsequent interviews. The topic guide was trialled with two patient representatives from the PPIE group prior to any participant interviews. Participants did not have access to the topic guide prior to the interviews but could access a copy if they wished to review questions at the end of the interview.

### 2.4 Participants

Purposive sampling was used to recruit PwL and family diverse participants to ensure collection of data rich information (Patton, 2002) from participants who were knowledgeable about the study question (Cresswell Jw, 2011). The sampling strata at the outset included mix of gender for both PwL and family members that is, some male family members and females with a laryngectomy; from a range of timepoints post‐surgery, mix of working age and retired participants from a diverse range of backgrounds for example, some living alone.

Family member sampling included formal and informal carers for example, close friends. The approach to purposive sampling was monitored and reviewed against the purposive strata set at the outset. As such, following fourteen interviews, sampling was targeted to females with a male family member, those living alone, and those of working age or who were employed.

Participants were recruited from three head and neck cancer centres in the United Kingdom: South Tyneside and Sunderland NHS Foundation Trust, The Newcastle‐upon‐Tyne Hospitals NHS Foundation Trust and Liverpool University Hospital NHS Foundation Trust. These settings provided sufficient access to potential participants to achieve recruitment targets, whilst also ensuring access to sample a diverse population, aiming to be representative of the national laryngectomy caseload. Strategies for recruitment of PwL participants were extensively discussed with PPIE representatives beforehand, to ensure these would be acceptable to patients: for example, suggestions to incorporate email or text response to the invitation letter to increase the pool of potential respondents.

PwL and family member participants were recruited via clinicians at each participating site. The clinicians identified potential participants using the study eligibility criteria. All PwL along with their family members who were emotionally and physically stable in the community and over 18 years old were eligible to participate in the study. People were excluded if they did not have sufficient capacity to provide informed consent to the study: for example, people living with advanced dementia.

Clinicians identified potential participants via review of their laryngectomy caseload lists where they could access basic patient demographics including telephone numbers or email addresses. Initial contact was made either in person or via contact details with eligible PwL and/or their family members about participation in the research study. Recruiting clinicians either asked PwL whether they would like to invite a family member to participate in the study with them or approached family members alongside PwL if participants were approached in‐person for example, in clinic or at a laryngectomy group.

### 2.5 Data Generation

Participants were offered the option of individual or dyadic interviews taking place virtually (via teleconferencing platforms like Microsoft Teams) or in‐person, at their preferred location depending on their needs. As a result, some participants were interviewed in their own home, some in clinical settings and others via telephone or online. Interviews were scheduled at the participant's preferred time: for example, around school times to accommodate participants who had caring responsibilities for school‐aged children. All PwL participants used a range of alternative communication methods: for example, silent articulation, writing, electro‐larynx. Specialist facilitation was provided on an individualised basis, to ensure all eligible participants who wished to participate were able to.

Interviews were conducted by the lead author (xxx), an experienced speech and language therapist with expertise in laryngectomy care, and doctoral student with formal training and practical experience in qualitative research methods. Some participants had an established relationship with the researcher due to previous clinical intervention provided; however, the role of the lead author within the interview setting was clarified as a researcher, not as a clinician, with those participants. All participants had knowledge of the lead author's clinical background from the participant information sheet.

Informed written or audio consent was taken prior to the interviews. All participants completed a baseline self‐reported demographic questionnaire prior to the interview. Interviews lasted 52 min on average and were digitally recorded using a digital voice recorder, with field notes taken. For participants interviewed as a dyad, each question was asked to the PwL and the family member individually to allow both participants time and space to have their views and experiences heard. Although not required, provisions outlined in the study's distress protocol were available should participants exhibit signs of distress during the interview. These included recommended prompts to assess participant well‐being and determine the appropriateness of continuing the interview process. All participants were offered the opportunity to de‐brief after the interview and provided with a de‐brief sheet which included sources of further support if needed. The lead author kept a reflective written diary following each interview which was reviewed prior to the next scheduled interview.

Interviews were transcribed verbatim, initially by the lead author (xxx) for familiarisation, and then by a secure transcription service affiliated with the University of Liverpool. Interviews were stored securely in a password‐protected folder on The University of Liverpool's secure servers, which only named members of the immediate research team could access. A pseudonym, which participants had the option of choosing, was used for all participants at point of data transcription. Interview recruitment/sampling was stopped once theoretical sufficiency was judged to have been reached (Clarke and Braun, 2013).

### 2.6 Analysis

Braun & Clarke's reflexive approach to reflexive thematic analysis (Braun and Clarke, 2008; Clarke and Braun, 2013) was used to conduct in depth data analysis. An inductive, semantic and realist approach to data analysis was taken which, as per Braun & Clarke (Clarke and Braun, 2013), meant working with the data from a ‘bottom up’ approach. This enabled the researcher to deeply look at the participants’ perspectives as well as the context of the data, focusing analysis on an assumed reality evident in the data, to ensure that the themes explicitly represent the content of the data set. This approach allows for a rich, thematic analysis of all the interview data with the final themes providing an insight into a topic which to date, has been under‐researched (Braun and Clarke, 2008). To achieve this, the six recursive steps as described by Braun & Clarke (Braun and Clarke, 2008) were used: familiarisation, coding, generating initial themes, reviewing themes, defining and naming themes and writing up.

The lead author (xxx) listened to all interview audio‐recordings alongside the transcripts to support deeper data contextualisation (Byrne, 2021), read the interview transcripts a minimum of three times, coded the data using NVIVO software (v. 14) and generated initial themes. This was done on an interim basis after every 3–4 interviews to ensure an iterative approach to data collection and analysis was taken.

The lead author undertook line‑by‑line coding of each interview transcript, assigning codes to all data segments considered meaningful. For dyad interviews, participants’ responses were transcribed separately to enable individual line‑by‑line coding. These codes were developed and refined throughout the analysis process as the lead author reviewed the interview transcripts. For example, codes that had the same definition or meaning were combined. The lead author then moved into grouping these codes together to search for initial themes using a creative and active approach (Clarke and Braun, 2013): the lead author went back and forward between the groups of codes, moved ideas around and became an active participant in the development of the initial themes. The research question was also actively considered throughout the analytic process. Data from dyad interviews were not analysed separately from individual interviews, reflecting the collective focus of the study rather than distinguishing between interview formats. The codes and initial themes were discussed with the research team, PPIE group and healthcare professional advisory group again on an interim basis, to ‘sense‐check’ and provide richer data interpretation (Byrne, 2021; Clarke and Braun, 2013). Following this, analysis moved into defining and naming final themes. All participants were provided with a summary of the final themes to comment on prior to final naming. Two participants provided feedback, and all agreed with analysis. No changes were made to the themes.

### 2.7 Reflexivity

The lead author is a speech and language therapist with specialist expertise in laryngectomy and experience in service development and pathway improvement. This professional background informed aspects of the research process, particularly the interpretation of data, and was addressed through ongoing reflexive practice.

### 2.8 Ethical Approval

Ethical approval for the study was provided by XXX. All participants received a participant information sheet outlining the study. This was provided by the clinicians responsible for recruitment. Participants were given the opportunity to ask questions prior to providing either informed written or audio consent to participate. Questions and informed consent were taken by the lead author. To note, for those consenting via audio, the lead author read each item on the consent document to the participant and asked them to verbally confirm their understanding and consent. Where participants were known to the lead author through clinical practice, care was taken to explicitly clarify prior to informed consent that participation was voluntary and separate from the therapeutic relationship, and that decisions regarding participation would not affect clinical care.

## 3 Results

Thirty‐six PwL were screened for eligibility. Thirty‐three PwL were eligible to participate, of whom seven declined for various reasons including being a ‘private person’ or not feeling psychologically able to participate. Seventeen PwL and thirteen of their family members participated in the interviews. Of these, eleven were dyad interviews (i.e., PwL and their family member together), six were PwL only and two included family members only, resulting in nineteen interviews in total. Twelve were in‐person interviews and seven were virtual for example, telephone or Microsoft Teams. In two individual interviews with PwL, family members were present but did not participate and are therefore not counted in the family member participants. Nine PwL and five family members had a previous clinical relationship with the researcher.

The majority of PwL were White British males (*n* = 13). PwL participants had an age range of 39–88 years old, mostly lived in areas with higher levels of social deprivation with secondary school education the highest level completed. All bar one participant was either retired or unemployed. The length of time since laryngectomy was variable ranging from <6 months to 5–10 years. Participants used a range of communication methods including electrolarynx, silent articulation and writing; however, most participants used trache‐oesophageal voice (*n* = 12). Distance from the treating centre ranged from 1.5–24.5 miles with the average distance being 10 miles.

Ten female and three male family members took part, (age 53–83 years old). Most family members were from the most socially deprived areas, had a secondary school education level and were retired. All family member participants were spouses living with the person with a laryngectomy and had a relationship with that person of more than 21 years.

**FIGURE 1:**
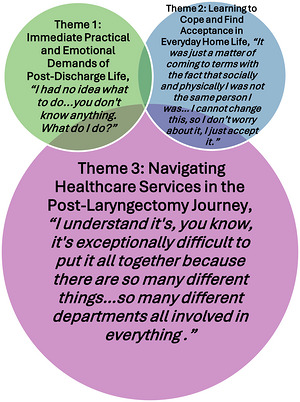
Final themes.

Three inter‐related themes were established following data generation and analysis. Figure 1 shows these themes, each with a quote to demonstrate the central organising concept of the theme: Immediate Practical and Emotional Demands of Post Discharge Life (theme one), Learning to Cope and Find Acceptance in Everyday Home Life (theme two), Navigating Healthcare Services in the Post Laryngectomy Journey (theme three). Themes one and two reflect the lived experience after laryngectomy in the early phase and longer‐term coping and adjustment trajectory. Theme three is an integrative theme describing how healthcare services shape these experiences for PwL and their families. All themes are integrated with one another, reflecting the central relevance to lived experience.

### 3.1 Theme one: Immediate Practical and Emotional Demands of Post‐Discharge Life, ‘I had‐ no Idea‐ What‐ to do‐…You Don't Know Anything. What do I do?’

This theme focuses on both the practical and emotional responses to life at home immediately after discharge, from the perspectives of PwL and their spouses. The practical demands of laryngectomy management are inextricably linked to the emotional load of adjustment and acceptance, with each shaping how PwL and their families navigate the early post‐discharge period.

PwL and their families talked about the emotional and practical response to life at home particularly within the early days and weeks post‐discharge. For most people, the initial period at home was marked by a sense of trepidation, with some describing fear about not providing care correctly, expressing concerns about safety, particularly around breathing and communication, *‘she went from saying, I want to go home, I want to go home, get home then you're scared when you're home…because she's breathing through a hole in her neck’* (Alex, spouse).

People expressed negative feelings around this transition which they linked to uncertainty around adjusting to the practical and functional changes of living with a laryngectomy. These encompassed living with changes to communication, *‘Nervous to go home to start with…all the unknown and the panic and the worry of it…it was mainly that I couldn't speak’* (Louise); and changes to appearance, *‘It has been very difficult afterwards; I find it embarrassing [referring to the stoma].’* (Anthony, PwL). Similarly to PwL, some spouses also experienced this strong emotional response to life after laryngectomy. This response was particularly stark in the spouses who take on some of the caring responsibilities, with all reporting they felt unprepared for what life would be like after laryngectomy, *‘We had no idea what to do and where to go, you're on your own*’ (Samantha, spouse).

Many also felt overwhelmed by the volume of equipment and the responsibility of independently and safely managing the airway without immediate access to specialist healthcare support, *‘There was a big box delivered, and I had no idea what it was. I didn't know that that would be there like that. And it was three weeks before we found out what that was.’* (Betty, spouse). People also start to consider the practicalities of accessing and managing the equipment needed to care for and maintain the laryngectomy stoma, *‘You've got to get a prescription from your doctor, and it seems to take forever…they need to make it easier to do the ordering.’* (Liz).

### 3.2 Theme two: Learning to Cope and Find Acceptance in Everyday Home Life, ‘It was Just a Matter of Coming to Terms With the Fact That Socially and Physically I was not the Same Person I was… I Cannot not Change This, so I Don't Worry About It, I Just Accept It.’

This theme moves beyond the immediate post‐discharge period to consider the longer‐term survivorship journey, highlighting how experiences and needs evolve well after the initial adjustment phase, *‘I think on the whole it's been a matter of time, really, and coming to terms and beginning to know exactly what the various materials will do’* (John).

For some individuals, especially men living alone following their laryngectomy, there were pronounced and enduring feelings of anxiety and depression into the survivorship phase of laryngectomy. Limited confidence and competence in communication appeared to play a significant role in sustaining these emotional challenges, *‘I was getting a bit depressed, I just said, I don't want to live like this, you know? I don't know if it was because I didn't have the voice, or‐ I didn't want to go out, I felt really down.’* (Gavin). For others, the emotional challenges were not highlighted as much, but this could be because many spouses took this on, sidelining their own emotional and practical needs, often for extended periods, as their attention became centred on supporting the person with a laryngectomy. *‘I just didn't know if I could do this or not. But obviously, you push yourself and you push yourself. And you just deal with however it's affecting you; you've just got to put it to one side and deal with it later.’* (Diane, spouse).

However, for years after their laryngectomy, people continued to describe persistent fears about stoma safety, particularly in the context of emergencies. Communication challenges amplified these concerns, suggesting that this sense of vulnerability may endure throughout survivorship, *‘What do we do because you can't ask for help and this has always played on my mind… what happens if I'm not here?*’ (Bob, spouse).

For some PwL and their spouses, access to support groups and voluntary services supports with confidence, emotional adjustment and acceptance to life after laryngectomy, *‘They brought me out of my shell a bit more. You can just go there and talk away. If I didn't get there, I would've been a home person, I would never have got out.’* (Percy). However, not everyone places the same amount of value on these groups or services, but there is limited rationale as to why this is the case for those participants, *‘We've never, I would say we've never been one for accessing any support groups actually in all the time that we you've been going through it.’* (Danielle, spouse).

### 3.3 Theme Three: Navigating Healthcare Services in the Post‐Laryngectomy Journey, ‘I Understand It's, You Know, It's Exceptionally Difficult to Put It all Together Because There are so Many Different Things…so Many Different Departments all Involved in Everything’

This theme represents the largest and most integrative element of the analysis, examining how healthcare services shape life after laryngectomy for individuals and their families across the entire post‐discharge trajectory. Overall, when support was available and able to meet the needs of PwL in the community, it provided a sense of being cared for. These interactions and relationships were characterised by trust and a sense of their laryngectomy care needs being fully understood and prioritised. Additionally, these interactions were very personal, with people feeling that the healthcare professional knew them as an individual, *‘The chemist [pharmacy] have been brilliant…honestly the staff there are lovely. As soon as I walk in, they are like ‘Ah Percy, you alright?’.’* (Percy).

However, the opposite was also true: people felt destabilised by a system that was absent, particularly in the early‐period post‐discharge *‘It was just very daunting after initially being discharged…no one came out to see me.’* (Mandy); or by a system which appeared uncertain about how to support them, *‘They (GP) don't know me from Adam, simple as. And they do not know what is going on down here (stoma). They don't even say, ‘take your tube out so I can have a look’. They have never seen in there…how would they know what they are looking for?’* (Percy); overall impacting on feelings about safety at home, *‘I don't feel safe.’* (George). In the absence of adequate input from healthcare professionals, spouses and carers often stepped into this role, particularly due to feelings around communication competence from PwL, *‘Like ringing up me GP, like I said I get me sister to do it because it's like, I kinda feel like I'm embarrassed by me voice, you know what I mean?’* (Louise), even if this impacted negatively on the spouses/carers, *‘I'll struggle on and do what I can myself. And if it runs me into the ground, it runs me into the ground, but I feel it is my job to look after him…that's the way I've felt… without the help…if I hadn't have been there to do that for him, I don't know where that care would have come from.’* (Mary, spouse).

In relation to the system itself, all PwL perceived that services from healthcare professionals were sparse, not timely and/or not in the right place, *‘Why aren't the districts trained up on that [laryngectomy]? I just found that really ridiculous.’* (Diane, spouse). In general, people described uncertainty about who to contact for specific issues, which reflected a broader sense of being lost within a system that lacked clarity and clear pathways, *‘That's how we're just finding our way round the systems now, because we've never used them…another problem is the <place> is in a different health authority to <town>. And it seems as if like ne'er the twain meet up.’* (Betty, spouse). People therefore defaulted to contact with their specialist centre as it was felt that they would be most likely to have the knowledge and care to support them, *‘I wouldn't have contacted my doctors, I would have contacted the head and neck specialists before I went to my doctors.’* (James).

As a result, the absence of personalised interventions following discharge appeared to negatively affect individuals’ emotional wellbeing and functional adjustment to life after laryngectomy, *‘I think he should have had more speech therapy once he got home. I feel like he could have done with more. I know that he was sometimes a bit embarrassed about trying to talk even though he was trying to do it.’* (Diane, spouse). Additionally, navigating practical challenges longer‐term had a noticeable impact on emotional wellbeing, *‘I see Mandy get frustrated when the delivery comes and it's the wrong stuff, you know? [2–3 years after laryngectomy]’* (Steve, spouse).

Participants did problem‐solve some of these issues by talking about how healthcare services could be integrated and personalised to meet the needs of individuals. Specifically, people want input from both specialist and community services but this needs to be in the right place for that individual depending on their healthcare, social and financial needs, *‘I live in <name> and I have to get two buses cos I don't drive. Two buses there, two buses back.…that's four buses I have to get to get to the hospital…so it becomes quite a few hours out of the house, you know especially in the winter weather, so you know it's not ideal really, so home visits I suppose would be easier…’* (Louise). For services to be truly integrated, participants believed that primary and community care staff required better training, *‘They need to know the difference between the and what's it like…a laryngectomy and a trache. Then this is permanent. This is no going back. It's not a chance of it. It's not a case of you might have it for six months or a year. You've got it for life.’* (Jack). They reported that, with improved staff knowledge and skills, PwL and their families would be more likely to access and place trust in these services, which in turn could reduce feelings of being unsafe at home following hospital discharge, *‘The district nurse would have been great if she had knowledge to answer the questions that we were asking.’* (Liz).

## 4 Discussion

### 4.1 Analytic Conclusions Across Themes

To our knowledge, this is the first qualitative interview study with PwL and their family members specifically focussing on their lived experience of healthcare services following discharge at a range of time‐points post‐surgery. Three inter‐related themes were generated from thematic data analysis: ‘Immediate Practical and Emotional Demands of Post‐Discharge Life.’; ‘Learning to Cope and Find Acceptance in Everyday Home Life.’; and Navigating Healthcare Services in the Post‐Laryngectomy Journey.’—the over‐arching theme which integrates the experiences described in the earlier presented themes. Collectively, these themes highlight the significance of immediate practical and psychological adjustment in the early stages at home with an identified greater need for health and social care intervention. However, the health and social care intervention received is perceived to be challenging by PwL and their families due to the variable experiences of healthcare services after laryngectomy, most often linked to those services provided in community or primary care settings. This is further exacerbated by their experience of accessing care across services within what is felt to be a disjointed healthcare system. Consequently, difficulties in accessing and navigating healthcare services after laryngectomy affect how PwL and their families adjust to and accept life at home. Improved experiences of health and social care interventions could help PwL and their families move out of the immediate practical and emotional demands stage to reach the adjustment and acceptance stage of recovery sooner within the post‐discharge laryngectomy pathway.

First and foremost, PwL and their families want specialist services for their laryngectomy care from specialist teams in the right place for them. In the current study, some PwL and their families showed an openness to local or domiciliary care post‐hospital discharge and beyond, but only when this was provided by specialist teams or by healthcare professionals whom they perceived as having the required knowledge and skills. This view may be common to all head and neck cancer patients (Checklin et al., 2025) to ensure that their supportive care needs are met, however, people with a laryngectomy have specific care needs linked to safety concerns around airway management and communication changes. Additionally, Dawson's study (Dawson et al., 2019) explored the acute experiences of care for people following surgical treatment for head and neck cancer. Although PwL were not included in this study and the focus was on the acute part of the pathway, this study highlights the importance of the care environment that is, being linked to where people trust staff, namely the hospital setting. This is likely to be because strong therapeutic relationships have been built within the acute inpatient care phase with people often reporting that they feel heard, understood and listened to by healthcare professionals within this environment (Dawson et al., 2019). It is possible that PwL are still psychologically in this acute care phase in the early few weeks and months at home, having not yet transitioned into the next phase of their recovery (Dawson et al., 2019; Dawson et al., 2019). As such it is important to prepare PwL and their families for this transitional phase before it happens that is, whilst still in hospital; with healthcare professionals from both the specialist centre and primary care/community services. In doing this, it is more likely that PwL and their families will access the most appropriate services for their needs, reducing the dependence on staff from the specialist centres for non‐specialist care needs. This is further supported by our previous work (Watson et al., 2022) which highlights the difficulty in transitioning from hospital to home, and along with the findings from this study, advocates for more support in community settings. Providing more support in the community could positively impact on the adjustment back to a ‘new normal’ from both a functional and emotional perspective for both the PwL and their families. However, local service models and staffing variations must be considered to ensure contextually appropriate implementation for the local population.

The findings here further demonstrate that people and their families want non‐laryngectomy specialist healthcare professionals (for example, district nurses and GPs) to understand laryngectomy and what has happened to them. Furthermore, PwL and their families also want non‐laryngectomy specialist healthcare professionals to understand what having a laryngectomy means for them. This encompasses the functional and practical implications of living with a laryngectomy as well as the psychological and social adjustment and acceptance to a new way of life. However, the data reported here demonstrate that the experience of community services for most PwL and their families is poor, leaving PwL and their families feeling under‐supported by healthcare professionals in the community. Participants also perceived that community staff lack the required laryngectomy knowledge and skills. These findings are consistent with previous research (Bickford et al., 2019; Watson et al., 2022), which also identified challenges in relationships with non‐laryngectomy specialist healthcare professionals, particularly when those healthcare professionals lacked laryngectomy‐specific knowledge and skills (Watson et al., 2022). Furthermore, while participants in previous studies (Bickford et al., 2019; Watson et al., 2022) were no more than two years post‐laryngectomy, several individuals in the current study were between five‐ and ten‐years post‐laryngectomy and continued to report persistent challenges related to healthcare service provision and the laryngectomy‐specific knowledge and skills of professionals in primary and community care. Similarly, Antin's study (Antin et al., 2021) reported that only 43% of participants felt that community healthcare professionals were adequately equipped to meet their needs. This study has further extended this finding with participants highlighting the need for non‐laryngectomy specialist healthcare professionals to better understand their needs from prior to discharge to support in the immediate adjustment phase at home, to the long‐term management of laryngectomy including issues around ageing with a laryngectomy. This could help PwL and their families to feel more confident accessing the services that non‐laryngectomy specialist healthcare professionals provide, ensuring that their needs are met life‐long. To do this, bespoke education and training focussing on community care needs to be developed (Watson et al., 2025). If this shift was made, there would likely be a reduction in dependency on the specialist centres for intervention which does not need to be provided there, for example, specialist staff providing long‐term equipment supplies. This would support the move towards greater provision of care closer to home (Darzi, 2024), a key driver in the NHS long‐term plan (Alderwick and Dixon, 2019).

However, to successfully achieve this, communication channels and collaborative working between primary, secondary and community services must be better. PwL and their families in this study have highlighted poor communication and a lack of collaboration between services. Participants highlighted how this results in unintended consequences, for example., over‐use of secondary care and unmet needs of family members; as well as adding to distrust in more general services due to the lack of information received from acute services. Similar challenges have been demonstrated in Foley's study (Foley et al., 2022), in which participants from rural areas of Australia highlighted the challenges in navigating a complex post‐treatment pathway across multiple services, specialities and healthcare professionals, resulting in higher levels of emotional distress and unmet care needs. If the future of healthcare services is focused on more community‐based, neighbourhood models of care (Darzi, 2024), there needs to be a shift in laryngectomy services towards a multi‐disciplinary approach that spans across teams and settings, reflecting the global emphasis on integrated and patient‐centred care.

The extra stress and concern for PwL and their families caused by lack of care coordination and poor communication, adds more pressure on family members to support navigation of services. This is supported by other qualitative research (Brehmer et al., n.d.; Checklin et al., 2025; Offerman et al., 2015) which highlights the psychological and social impact that the lack of support has on families, such as changes to social interactions and communication and intimacy with partners (Dooks et al., 2012), with a heightened emotional response in the carer population (Hanly et al., 2016; Rogers et al., 2024) particularly in the first three months following treatment (Patterson et al., 2013). The need for support from family members is particularly necessary, especially for PwL who are unable to, or do not feel confident in, communicating with healthcare professionals, particularly when telephone contact is the first port of call with many service providers. Ultimately, it should not solely be the family members’ responsibility to navigate services as they themselves are experiencing a significant adjustment to life after laryngectomy, and other solutions, such as remote care within a hybrid pathway as trialled by van den Besselaar (Van Den Besselaar et al., 2025) with people with a palliative head and neck cancer diagnosis, could be explored. Participants in van den Besselaar's (Van Den Besselaar et al., 2025) study felt satisfied with access to remote care monitoring and could also support healthcare professionals to optimise time spent in face‐to‐face consultations, thereby reducing over‐use and increasing productivity in specialist settings (Alderwick and Dixon, 2019). However, caution should be applied here as some PwL and their families report poor access or use of digital solutions, and implementation could widen the health inequalities gap within a population with known low health literacy levels (Nilsen et al., 2020) and from some of the most socially deprived areas (Simpson et al., 2020).

Another potential solution to improve post‐discharge laryngectomy care is a model to link healthcare services and community programmes together through strategies such as resource sharing, clear communication and co‐ordination of staff and services (Alsbury‐Nealy et al., 2022). Foley's research (Foley et al., 2022) similarly advocates for strengthened local care teams, emphasising that shared care arrangements can reduce service inequities and enhance the overall healthcare experience for people following head and neck cancer treatment. Together, this evidence highlights the need for integrated, collaborative approaches that bridge gaps between services and support more consistent, person‐centred care after discharge. As well as reducing the burden on family members, a solution such as this could also support integration of services outside of healthcare alone. Some PwL and family members in this study have highlighted the need for increased input from social and voluntary sectors to meet their wider psycho‐social needs, but these services are currently stark in laryngectomy pathways. Bar laryngectomy peer support groups, which PwL and their families have varying views and experiences of; intervention beyond the healthcare needs of laryngectomy does not appear to fully be in place. The link between sectors is also a key ambition in the next ten years for the health service, supporting the move towards a neighbourhood NHS (Alderwick and Dixon, 2019; Darzi, 2024). It is therefore vital that laryngectomy services begin to make changes in this direction.

Finally, it is important to acknowledge that whilst there are similarities in the experiences of participants in this study, everyone is individual and nuances in the data support the need for care to be provided in that way. For example, some people in the study described a need for more emotional support, particularly the males who lived alone; whereas others felt they needed more support in functional rehabilitation, and some people wanted very practical input. These needs also may change, particularly for those who have been living with a laryngectomy for a long time and are considering age‐related changes that is, dexterity and vision issues which would impact on stoma and voice‐prosthesis care, potential loss of their spouse which may impact on travel, laryngectomy care and navigation of services. People at this stage start to think about what and where laryngectomy services could be provided to support their needs. This supports the need for all PwL to have access to personalised cancer care to ensure that their individual physical, practical, emotional and social needs are fully met (Alderwick and Dixon, 2019; Le Boutillier et al., 2023).

### 4.2 Implications/Future Directions for Research

Although to our knowledge, this is the only qualitative research study specifically exploring the lived experience of community care following laryngectomy with both the person and their families, there are areas which should be considered for further exploration in future research. This study has highlighted a potential need for those living alone, particularly males, to have increased support at home in the early weeks and months post‐hospital discharge. Clinicians working in specialist centres could be more aware of this demographic early in the laryngectomy pathway to help tailor intervention to this population. However, further research is required to explore this area in more depth, mainly because no females living alone participated in this study. As such, interviewing males who live alone and females who live alone would help to understand this area more. This could also be combined with looking into the differences in coping styles between genders, age and social status.

Collaboration of services across community, primary and acute care has been flagged as an area which requires development and practical solutions to ensure that the needs of PwL and their families are fully met. Clinicians working within specialist units could seek to establish improvements in communication with local primary and community care teams at the point of discharge to help support the transition from hospital to home. Linked to this, further research could explore how the laryngectomy pathway may look across different time‐points for example, pre‐surgery, acute inpatient stay, acute post‐discharge, adjustment, long‐term rehabilitation, survivorship; to investigate how inter‐disciplinary services across all settings could be integrated and care experiences improved. Learning could be sought from other pathways which have developed these models, for example stroke (Langhorne et al., 2017).

Finally, there is a need from the perspective of PwL and their families for non‐laryngectomy specialist healthcare professionals to have better laryngectomy knowledge and skills so that they can provide vital care and support at home. Future research could explore how to co‐produce an intervention to support community healthcare professionals to upskill in laryngectomy care. An intervention such as this may better meet the short and long‐term health needs of PwL and their families.

### 4.3 Evaluation of the Research

While the participant sample reflects some geographical diversity, and findings have been shared with UK‐wide patient groups, as previously discussed, local service models and staffing variations must be considered when applying results.

The participant sample in this study lacked ethnic and gender diversity. The predominance of White participants likely reflects, in part, the geographical location of recruiting sites. Although evidence on the laryngectomy population is scarce, Delon et al. (Delon et al., 2022) identified higher cancer incidence within the White ethnic group in the UK. Nonetheless, future research should aim to include diverse groups to enrich and contextualize these findings, particularly because those from minority ethnic groups often report worse experiences of primary healthcare and cancer care (Damaree‐Cotton and Singh, n.d.).

Although the study inclusion criteria allowed for recruitment of a range of family members and informal carers, the only people recruited to the study were partners of PwL. As such, views of other groups such as children, parents and friends have not been explored. This again could be another area to further explore in future research, particularly as there appears to be early suggestions around unmet supportive care needs in this population.

Finally, all participants were recruited from specialist hospital centres as this is where management of laryngectomy services sits. However, there is potential that the views of some PwL may have been missed—specifically those who do not engage with specialist services following discharge. Additionally, healthcare professionals recruiting participants may have recruited those who have a particular perspective or experience of community services.

### 4.4 Research Process and Practices: My Role in Shaping Research/Reflexivity

The lead author (xxx) is a speech and language therapist with specialist expertise in laryngectomy and has a specific interest in service development and pathway improvement. This inevitably shaped aspects of the research process, particularly in data interpretation. Furthermore, the familiarity with two of the recruiting centres and prior clinical relationships with certain participants may have influenced the initial interpretation of data, given the inherent difficulty in fully disentangling the dual roles as researcher and clinician. To mitigate potential bias, findings were discussed iteratively with the wider research team (all co‐authors—xx, xx, xx, xx), as well as with healthcare professionals and patient and public involvement groups throughout data collection and analysis. These collaborative engagements helped ensure that interpretations were critically examined and not solely influenced by the perspective of the lead author.

Some participants from the centre where the lead author holds clinical sessions may have chosen to participate due to personal familiarity. On reflection, these individuals tended to share more speech and language therapy‐specific insights, likely shaped by the association to the professional role of xxx. Additionally, some participants referred positively to these previous clinical interactions with xxx. Upon recognising this, xxx made a conscious effort to redirect questioning towards the broader laryngectomy pathway to avoid over‐focusing on their own clinical area. In addition, participants interviewed in hospital settings, as opposed to their own home, occasionally shared information in a problem‐solving manner, reflecting typical clinical interactions within that environment. At these points, xxx paused the interview, clarified their role as a researcher, and, with participants’ consent, acknowledged that any clinical concerns raised would be handed over to appropriate colleagues for further follow‐up. This dynamic was also considered during analysis by discussion with all co‐authors to ensure balanced representation across the dataset. Discussions also occurred in regular academic supervision and with consultation with patient and public involvement groups to critically review the data interpretation to ensure that this information was not being seen through xxx's clinical lens. In addition, a reflexive diary was maintained during data collection to support ongoing evaluation of interview practice and its influence on the generation of data, for example, adapting interviewer responses to remain neutral and facilitate deeper exploration of participants’ accounts.

## 5 Conclusion

This qualitative interview study has explored the lived experience of healthcare services following discharge from the perspectives of PwL and their families. There is a greater need not just for community healthcare professionals to have basic laryngectomy knowledge and skills, but also for better collaboration between healthcare professionals and services to improve support and intervention for PwL and their families after their surgery. It is critical that this is achieved early in the laryngectomy pathway to facilitate trust between healthcare professionals, patients and their families which could, in the long‐term, lead to an improvement in patient experience.

## Ethics Statement

Ethical approval for the study was provided by Yorkshire & The Humber—Leeds West Research Ethics Service Committee in June 2024 (Reference: 24/YH/0074).

## Conflicts of Interest

The authors declare no conflicts of interest.

## Supporting information




**Supporting Information**: jlcd70275‐supp‐0001‐Suppmat.docx

## Data Availability

The data that support the findings of this study are available on request from the corresponding author. The data are not publicly available due to privacy or ethical restrictions.
