# Supplementary material for: ‘We Needed a Hell of a Lot More Support, the Emotional Side of It, the Physical Side of It. Every Side of It, We Just Didn't get It.’ A Qualitative Study Exploring the Lived Experiences of Healthcare Services Following Discharge for People With a Total Laryngectomy and Their Families
Source: Int J Lang Commun Disord. 2026 Jun 18;61(4):e70275. doi: 10.1111/1460-6984.70275 (PMC13280431; doi:10.1111/1460-6984.70275)
Supplement: Supplementary file 1 — Supporting Information: jlcd70275‐supp‐0001‐Suppmat.docx [file JLCD-61-0-s001.docx]

Topic guide:  patient and family member interviews

**Introduction**

1. Introduce self and thank for agreeing to participate
2. Participation is optional and you can stop the interview or decline to answer specific questions if you wish. It should last no longer than an hour.

**Confidentiality and consent**

1. The findings will be written up as part of the PhD and for publication but you will remain anonymous. Would you like to choose a pseudonym (alternative name) to be used for these purposes?
2. I would like to record the interview to help capture everything you say and also to evaluate the questions that I use. I may repeat some of your answers to make sure I have understood you. Everything you say will remain on a secure network at Liverpool University which can only be accessed by the research team. Is this okay with you?
3. Clarify that they understand the purpose and confidentiality of the research and that they are happy to partake.  Consent form signed.
4. Any questions.

**These questions are about general information, just giving me an idea of the background of your laryngectomy.**

(*) This is about your experience when you were discharged home from hospital after having your (your family members) laryngectomy – what jumps out for you? What were the low points? Was there anything that particularly went well?

Probe further dependent on participant response

*I’d now like you to think about the few days before you (your family member) came home from hospital after the laryngectomy – what was that like for you? (probe for level of preparation e.g. discharge information, including setting i.e., all in hospital or any home leave; materials to support info (local or external e.g., NALC/social media); meeting healthcare professionals from the community)*

How did you think you would cope at home after the (your family members) operation? Why?

Why did you feel like that? (probe dependent on response)

**Discharge**

***Probing questions if not enough info from first general question***

(*) Can you tell me a little bit about what healthcare services were provided to you following your discharge from hospital? Who provided them? How did they help/not help? What went well? What could be better?

(*) Can you tell me a little bit more about any other support you had during this time? Who provided this support? What was helpful/unhelpful about this? Was there anything that didn’t go so well? If so, why? (probe further dependent on patient/family member response)

(*) Just following on from the previous question, reflecting back – who do you think is important during this time? Why do you think this? And what role do they provide?

If you could change anything about this time, would you and what would you change? Why would you change this?

**Thinking in the longer term**

(*) Can you tell me a little bit about the longer term needs you might have after your laryngectomy? Are these different to the needs that you had immediately after your discharge home? *If just family present then adjust question*

(*) Where would you prefer to access your laryngectomy care and why? Who would provide this care and why? Both immediately post-discharge and in the long-term. *If just family present then adjust question*

What are your health and social care needs (as a patient and/or family member- think about work, family, social)? What services would best meet these needs and why? (*)

(*) What do you think is important for healthcare professionals in the community to know and why?

(*) How could we improve services after laryngectomy to better meet your needs as a patient and/or family member? Why do you think this? (note – support groups and peer to peer support – ? access)

**Experience**

1. How would you describe your overall experience? Specifically of that period of time when you first went home after your laryngectomy?
2. Was there anything that could be done differently? Why?
3. How did it make you feel? Why?
4. What went well for you? Why?
5. How did it make you feel? Why?

**Summarising and reflecting**

Just thinking about your discharge after your (your family member’s) laryngectomy – is there anything else you want to talk about?

Is there anything else you would like to add?
